# Dose–response association of Chinese visceral adiposity index with comorbidity of hypertension and diabetes mellitus among elderly people

**DOI:** 10.3389/fendo.2023.1187381

**Published:** 2023-05-12

**Authors:** Yongcheng Ren, Lulu Cheng, Ranran Qie, Minghui Han, Lingzhen Kong, Wei Yan, Zheng Li, Yiduo Li, Yicun Lei

**Affiliations:** ^1^ Institute of Health Data Management, Huanghuai University, Zhumadian, He’nan, China; ^2^ Affiliated Hospital of Huanghuai University, Zhumadian Central Hospital, Zhumadian, He’nan, China; ^3^ Jiyuan Center for Disease Control and Prevention, Jiyuan, He’nan, China; ^4^ Department of Cancer Prevention and Control, National Cancer Center/National Clinical Research Center for Cancer/Cancer Hospital, Chinese Academy of Medical Sciences and Peking Union Medical College, Beijing, China; ^5^ Department of Epidemiology and Biostatistics, School of Public Health, Nanjing Medical University, Nanjing, China

**Keywords:** Chinese visceral adiposity index, comorbidity, mediation analysis, elder people, insulin resistance

## Abstract

**Background:**

Chinese visceral adiposity index (CVAI) is a reliable indicator of visceral obesity, but little is known about the association of CVAI with comorbidity of hypertension (HTN) and diabetes mellitus (DM). This study aimed to explore the associations of CVAI with HTN-DM comorbidity, HTN or DM, HTN, and DM in elderly people and evaluate the mediating role of insulin resistance in the associations.

**Methods:**

A total of 3,316 Chinese participants aged ≥60 years were included in this cross-sectional study. Logistic regression models were used to estimate odds ratios (ORs) and 95% confidence intervals (CIs). Restricted cubic splines were applied to explore the dose–response associations. Mediation analyses were used to assess the mediating effect of triglyceride-glucose (TyG) index in the associations.

**Results:**

The prevalence rate of HTN-DM comorbidity, HTN or DM, HTN, and DM was 13.78%, 72.26%, 67.16%, and 18.88%, respectively. Linear associations between CVAI and HTN-DM comorbidity, HTN or DM, HTN, and DM were found, and ORs (95%CIs) were 1.45 (1.30–1.61), 1.39 (1.28–1.52), 1.36 (1.25–1.48), and 1.28 (1.16–1.41) for per SD increase in CVAI. Compared with quartile 1 of CVAI, the risk of HTN-DM comorbidity, HTN or DM, HTN, and DM increased 190%, 125%, 112%, and 96% for quartile 4. In addition, we found TyG index playing a key role in the associations of CVAI with HTN-DM comorbidity, HTN or DM, and DM.

**Conclusion:**

CVAI is linearly and positively correlated with HTN-DM comorbidity, HTN or DM, HTN, and DM. The potential mechanism is insulin resistance largely mediating the associations.

## Introduction

Hypertension (HTN) and diabetes mellitus (DM) are two important risk factors for cardiovascular disease and premature death worldwide (1, 2). With the increasing prevalence of HTN and DM and intensification of aging, HTN-DM comorbidity has become a serious public health problem, especially in Chinese population (3–5). Data from China Kadoorie Biobank (CKB) showed that the prevalence rate of HTN-DM comorbidity increased by 162% (2.72% vs. 7.12%) during 8 years of follow-up (6). China Chronic Disease and Risk Factor Surveillance (CCDRFS) in 2018 including 134,950 participants aged ≥45 years showed that the total prevalence rate of HTN, DM, and HTN-DM comorbidity was 46.0%, 19.5%, and 12.3%; the corresponding prevalence rate was 59.2%, 25.0%, and 17.7% for participants aged 65–75 years and 67.6%, 26.7%, and 20.6% for participants aged ≥75 years (7). Because of the high prevalence and low control level, identifying risk factors, early detection, and subsequent intervention in HTN-DM comorbidity is urgently needed.

Chinese visceral adiposity index (CVAI), like visceral adiposity index (VAI) for western population, was established to estimate visceral adipose tissue (VAT) for Chinese population and can predict metabolic disorders well (8, 9). Previous studies have reported positive associations of CVAI with HTN (10, 11), DM (12, 13), and cardiovascular disease (14, 15), which mainly focus on single disease. The effect of CVAI on HTN-DM comorbidity was unknown, and the dose–response association was unclear. In addition, a recent study including 59,429 described that both VAT and visceral to total adipose tissue (VAT/TAT) ratio increased with increasing age (16). Therefore, it is essential to assess the association between CVAI and HTN-DM comorbidity in elderly people. Considering insulin resistance as the common mechanism of HTN and DM (17, 18), evaluating the mediating role of insulin resistance in the associations would benefit understanding the internal mechanism the association between CVAI and HTN-DM comorbidity.

Hence, this study aimed to explore the dose–response association of CVAI with HTN-DM comorbidity, HTN or DM, HTN, and DM in elderly people and test whether the associations are mediated by insulin resistance.

## Methods

### Study population

During 2019–2020, a total of 5,068 participants aged ≥60 years were selected from 10 community health service centers. After excluding participants with unknown status of HTN and DM (n= 453), missing data for age, body mass index (BMI), waist circumference (WC), triglycerides (TG), and high-density lipoprotein cholesterol (HDL-C) levels (n= 1,449), 3,166 individuals were included to explore the associations of CVAI and HTN-DM comorbidity, HTN or DM, HTN, and DM.

### Data collection

Data on demographic characteristics (age, gender, marital status, and educational level), lifestyle factors (smoking, drinking, dietary habits, and physical activity), and medical history (anti-hypertensive medication, lipid lowering, and antidiabetic history) were collected by uniformly trained medical staff. Height, weight, WC, and blood pressure (BP) were measured, and all instruments were calibrated before use every time. Fasting blood was collected for fasting plasma glucose (FPG), TG, total cholesterol (TC), low-density lipoprotein cholesterol (LDL-C), and HDL-C. Quality control was conducted by establishing a detailed study protocol, using standardized questionnaire, training medical staff uniformly, calibrating instrument before use, and inputting information doubly.

Smoking was defined as smoking ≥100 cigarettes in their lifetime and drinking as drinking alcohol ≥12 times during the last year. Ideal diet was regarded as good mixture of meat and vegetables. Ideal physical activity was defined as more than 30 min of light and/or more than 20 min of moderate physical activity and/or more than 10 min of heavy physical activity per day for more than 5 years. BMI was calculated as weight (kg) divided by height (m) squared. Triglyceride-glucose (TyG) index, a reliable indicator assessing insulin resistance, was calculated as Ln (TG × 88.55 × FPG × 18/2) (both TG and FPG units in mmol/L) (19). CVAIs were calculated as follows (8):


$$\begin{array}{c} CVAI\;\left(men\right)\;=-267.93\;+\;0.68\;*\;age\;+\;0.03\;*\;BMI\;+\;4.00\;*\;WC\;\\ +\;22.00\;*\;Log10TG-16.32\;*\;HDL-C \end{array}$$

$$\begin{array}{c} CVAI\;\left(women\right)\;=-187.32\;+\;1.71\;*\;age\;+\;4.23\;*\;BMI\;\\ +\;1.12\;*\;WC\;+\;39.76\;*\;Log10TG-11.66\;*\;HDL-C \end{array}$$

### Outcomes assessment

HTN was defined as systolic BP (SBP) ≥140 mm Hg and/or diastolic BP (DBP) ≥90 mm Hg and/or use of antihypertensive medication (20). DM was defined as FPG ≥7.0 mmol/L and/or current treatment with anti-diabetes medication according to the Chinese guidelines for T2DM (21). HTN-DM comorbidity was defined as participant having both HTN and DM. Participants with HTN or DM were having at least one of HTN or DM.

### Statistical analyses

Continuous variables were described as median (interquartile range) and categorical variables as number (percentage). Wilcoxon two-sample test or chi-square test was used to test differences between men and women. Logistic regression models were used to estimate odds ratios (ORs) and 95% confidence intervals (CIs) of HTN-DM comorbidity, HTN or DM, HTN, and DM for per standard deviation (SD) increase and quartiles of CVAI. Model 1 was adjusted for age and gender; model 2 was adjusted for model 1 plus educational level, marital status, dietary, smoking, drinking, physical activity, and family history of HTN or DM; and model 3 was further adjusted for model 2 and TC, cardiovascular disease, and cancer. Restricted cubic splines (RCS) were used to explore the dose–response association of CVAI with the outcomes. Subgroup analyses were conducted to identify the consistency of the results across gender (men vs. women) and age (<75 years vs. ≥75 years) and to explore possible interaction by multiplicative model. Sensitivity analyses were conducted to test the robustness of the results by excluding participants with cardiovascular disease or cancer.

Mediation analysis was used to explore the mediating effect of TyG index in the associations between CVAI and HTN-DM comorbidity, HTN or DM, HTN, and DM by the PROCESS procedure in SAS v.9.4 (22). Mediated effect values and 95% CI were evaluated by a bias-corrected non-parametric percentile bootstrap method with 5,000 random sampling times. Statistical analyses were carried out in SAS V.9.4 (SAS Inst., Cary, NC) and R V.4.2.2 (23). A two-sided *p*-value <0.05 was considered statistically significant.

## Results

### Characteristics of study participants

The characteristics of the 3,316 study participants are shown in **Table 1**. Median age was 69.93 (7.46) years, and 42.46% were men. Compared with women, men were more likely to have higher proportion of marriage, higher education level, smoking, drinking, ideal diet and physical activity, higher level of WC, CVAI, TyG index, and lower level of TG, TC, HDL-C (all *p <*0.05). Among those participants, 457 (13.78%) had HTN-DM comorbidity, 2,396 (72.26%) had HTN or DM, 2,227 (67.16%) had HTN, and 626 (18.88%) had DM.

**Table 1:** Baseline characteristics of study participants.

	Total (n = 3316)	Men (n = 1408)	Women (n = 1908)	*p*-value
Age (years)	69.93 (7.46)	69.88 (6.85)	70.00 (7.85)	0.1440
Marriage (%)	3,108 (93.73)	1,349 (95.81)	1,759 (92.19)	<0.0001
High school or higher (%)	724 (21.83)	1,047 (25.64)	1,545 (19.03)	<0.0001
Smoking (%)	269 (8.20)	257 (18.37)	12 (0.64)	<0.0001
Drinking (%)	32 (0.97)	30 (2.13)	2 (0.10)	<0.0001
Ideal diet (%)	3,088 (95.40)	1,329 (96.87)	1,759 (94.32)	0.0006
Ideal physical activity (%)	70 (2.11)	39 (2.77)	31 (1.62)	0.0234
Family history of HTN or DM (%)	50 (1.51)	22 (1.56)	28 (1.47)	0.8857
WC (cm)	85 (11)	86 (10)	85 (10)	<0.0001
BMI (kg/m^2^)	24.77 (4.24)	24.73 (4.16)	24.77 (4.25)	0.5877
FPG (mmol/L)	5.28 (1.24)	5.29 (1.26)	5.28 (1.21)	0.9596
TG (mmol/L)	1.54 (1.17)	1.38 (1.07)	1.67 (1.19)	<0.0001
TC (mmol/L)	5.06 (1.55)	4.82 (1.48)	5.27 (1.56)	<0.0001
HDL-C (mmol/L)	1.5 (0.55)	1.44 (0.53)	1.55 (0.57)	<0.0001
CVAI	120.65 (41.06)	107.22 (43.6)	127.88 (34.22)	<0.0001
TyG index	8.79 (0.82)	8.67 (0.86)	8.86 (0.79)	<0.0001
HTN (%)	2227 (67.16)	943 (66.97)	1284 (67.30)	0.8457
DM (%)	626 (18.88)	258 (18.32)	368 (19.29)	0.4835
CVD (%)	231 (6.97)	97 (6.89)	134 (7.02)	0.8810
Cancer (%)	7 (0.21)	2 (0.14)	5 (0.26)	0.7060

Data are median (interquartile range) or number (%).

HTN, hypertension; DM, diabetes mellitus; WC, waist circumference; BMI, body mass index; FPG, fasting plasma glucose; TG, triglycerides; TC, total cholesterol; HDL-C, high-density lipoprotein cholesterol; CVAI, Chinese visceral adiposity index; TyG index, triglyceride-glucose index; CVD, cardiovascular disease.

### Dose–response associations of CVAI with HTN-DM comorbidity

**Figure 1:**
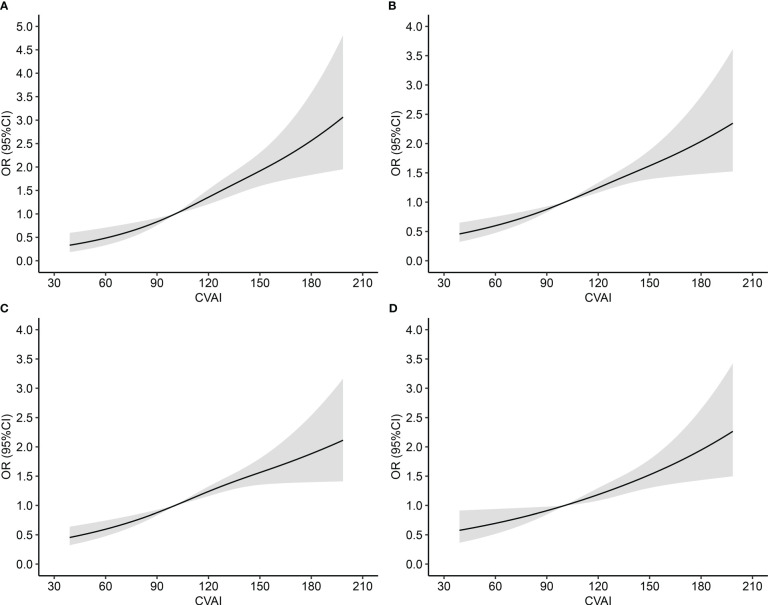
Dose–response association between Chinese visceral adiposity index and risk of comorbidity of hypertension and diabetes mellitus. OR, odds ratio; CI, confidence interval; CVAI, Chinese visceral adiposity index; HTN, Hypertension; DM, diabetes mellitus. **(A)** HTN-DM comorbidity; **(B)** HTN or DM; **(C)** HTN; **(D)** DM. Adjusted for age, gender, educational level, marital status, dietary, smoking, drinking, physical activity, family history of HTN or DM, total cholesterol, cardiovascular disease, and cancer.

**Figure 2:**
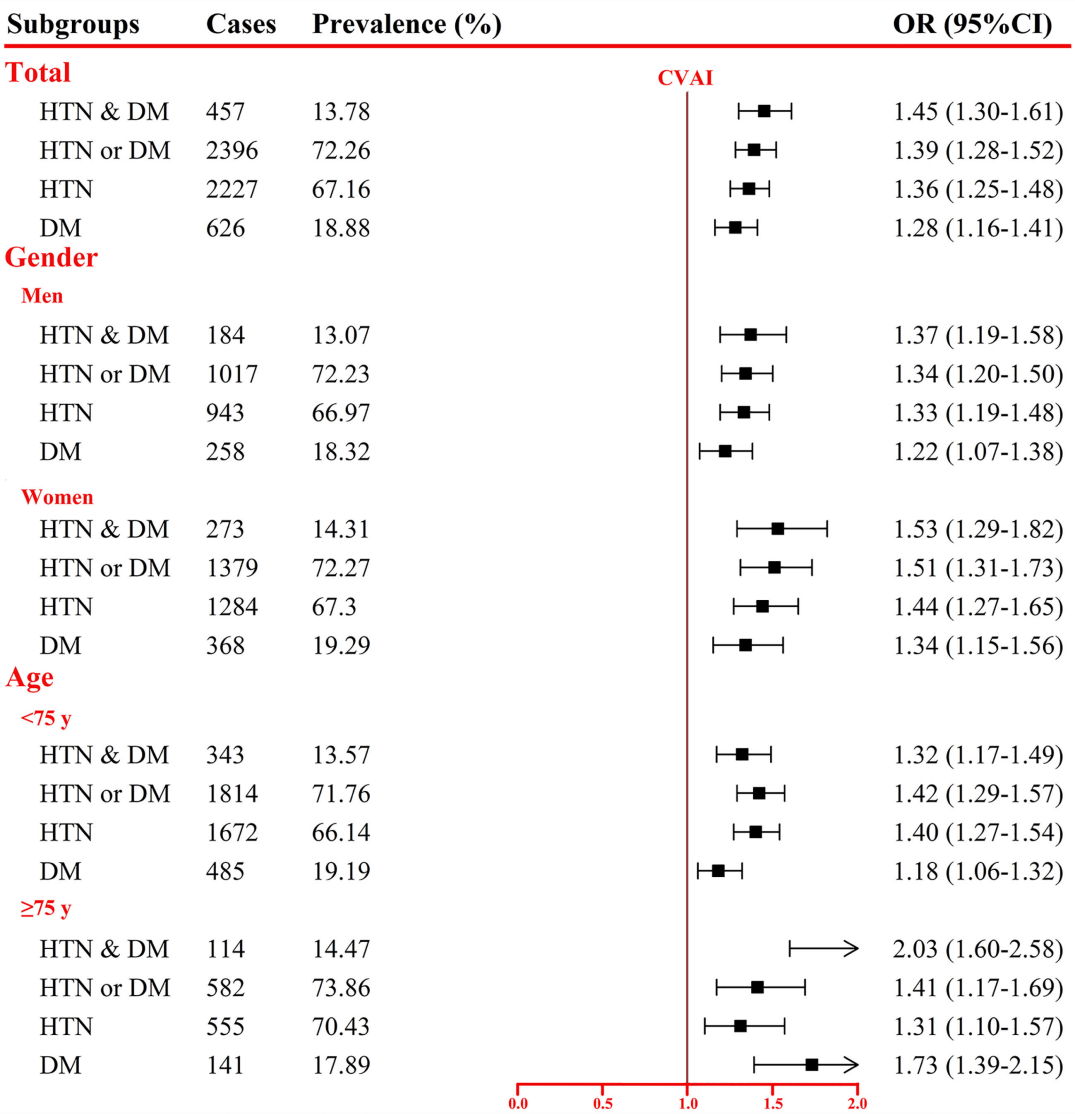
Association between per SD increase in Chinese visceral adiposity index and comorbidity of hypertension and diabetes mellitus. OR, odds ratio; CI, confidence interval; HTN, hypertension; DM, diabetes mellitus. Adjusted for age, gender, educational level, marital status, dietary, smoking, drinking, physical activity, family history of HTN or DM, total cholesterol, cardiovascular disease, and cancer.

RCS curve showed linear associations between CVAI and HTN-DM comorbidity, HTN or DM, HTN, and DM, with *p*-values of 0.2672, 0.3480, 0.1983, and 0.9019, respectively (**Figure 1**). After adjusting potential confounders, per SD increase in CVAI was associated 45%, 39%, 36%, and 28% increased risk of HTN-DM comorbidity, HTN or DM, HTN, and DM (**Figure 2**), and the corresponding ORs (95% CIs) were 1.45 (1.30–1.61), 1.39 (1.28–1.52), 1.36 (1.25–1.48), and 1.28 (1.16–1.41). The positive associations persisted on further excluding participants with cardiovascular disease and cancer (**Supplementary Table S1**).

In addition, we also assessed the associations stratified by gender and age (<75 vs. ≥75 years; **Figure 2**). Risk of HTN-DM comorbidity, HTN or DM, HTN, and DM associated with per SD increment in CVAI increased 37%, 34%, 33%, and 22% for men and 53%, 51%, 44%, and 34% for women. Participants aged ≥75 years had higher risk of HTN-DM comorbidity (103% vs. 32%) and DM (73% vs. 18%) than those aged <75 years (*p*_interaction_ *<*0.05). Per SD increase in CVAI was associated with 42% and 40% increased risk of HTN or DM and HTN for participants aged <75 years and 41% and 31% for those aged ≥75 years. Sensitivity analyses showed the similar results (**Supplementary Table S1**).

### Risk of HTN-DM comorbidity by quartiles of CVAI

After adjusting for the potential confounders (age, gender, educational level, marital status, dietary, smoking, drinking, physical activity, family history of HTN or DM, TC, cardiovascular disease, and cancer), compared with participants in quartile 1 (**Table 2**), the ORs (95% CIs) in quartiles 2–4 of CVAI were 1.57 (1.13–2.19), 1.97 (1.41–2.75), and 2.90 (2.11–4.00) for HTN-DM comorbidity; 1.32 (1.07–1.64), 1.58 (1.26–1.99), and 2.25 (1.77–2.86) for HTN or DM; 1.41 (1.14–1.74), 1.64 (1.31–2.04), and 2.12 (1.69–2.66) for HTN; and 1.16 (0.88–1.52), 1.38 (1.04–1.82), and 1.96 (1.50–2.56) for DM, respectively. With increasing CVAI quartile, risk of the outcomes increased substantially. Results of sensitivity analyses showed consistent associations (**Supplementary Table S2**).

**Table 2:** Risk for comorbidity of hypertension and diabetes mellitus by quartiles of Chinese visceral adiposity index.

	Quartile 1 (<100.07)	Quartile 2 (100.07–120.65)	Quartile 3 (120.65–141.13)	Quartile 4 (≥141.13)	*p-*value
HTN-DM comorbidity					
Cases	70	104	121	162	
Model 1	1.00	1.59 (1.15–2.20)	1.95 (1.41–2.70)	2.79 (2.04–3.82)	<0.0001
Model 2	1.00	1.57 (1.13–2.18)	1.94 (1.39–2.70)	2.84 (2.07–3.91)	<0.0001
Model 3	1.00	1.57 (1.13–2.19)	1.97 (1.41–2.75)	2.90 (2.11–4.00)	<0.0001
HTN or DM					
Cases	538	583	611	664	
Model 1	1.00	1.33 (1.08–1.65)	1.61 (1.29–2.01)	2.25 (1.78–2.84)	<0.0001
Model 2	1.00	1.34 (1.08–1.66)	1.60 (1.28–2.01)	2.26 (1.78–2.88)	<0.0001
Model 3	1.00	1.32 (1.07–1.64)	1.58 (1.26–1.99)	2.25 (1.77–2.86)	<0.0001
HTN					
Cases	484	547	573	623	
Model 1	1.00	1.44 (1.17–1.76)	1.68 (1.36–2.08)	2.19 (1.76–2.73)	<0.0001
Model 2	1.00	1.43 (1.16–1.76)	1.68 (1.35–2.09)	2.20 (1.75–2.75)	<0.0001
Model 3	1.00	1.41 (1.14–1.74)	1.64 (1.31–2.04)	2.12 (1.69–2.66)	<0.0001
DM					
Cases	124	140	159	203	
Model 1	1.00	1.17 (0.90–1.53)	1.41 (1.08–1.85)	1.97 (1.52–2.56)	<0.0001
Model 2	1.00	1.18 (0.90–1.55)	1.41 (1.07–1.85)	2.02 (1.55–2.64)	<0.0001
Model 3	1.00	1.16 (0.88–1.52)	1.38 (1.04–1.82)	1.96 (1.50–2.56)	<0.0001

Data are odds ratios (ORs) and confidence intervals (CIs).

HTN, hypertension; DM, diabetes mellitus.

Model 1: Adjusted for age and gender; model 2: adjusted for model 1 plus educational level, marital status, dietary, smoking, drinking, physical activity, and family history of HTN or DM; model 3: adjusted for model 2 and total cholesterol, cardiovascular disease, or cancer.

### Mediating effects of TyG index on association of CVAI with HTN-DM comorbidity

Results of the mediation analysis for TyG index is shown in **Table 3**. After accounting for potential confounders, CVAI was found positively associated with TyG index (path a), and TyG index associated with HTN-DM comorbidity (path b; OR, 3.18; 95%CI, 2.63–3.83). The total (path c) and indirect (path ab^#^) effects were statistically significant, and direct (path c’) effect was marginally significant, with adjusted OR (95%CI) of 1.45 (1.30–1.62), 1.32 (1.25–1.40), and 1.13 (1.00–1.27), respectively. TyG index largely mediated the association between CVAI and HTN-DM comorbidity.

**Table 3:** Mediating effects of TyG-mediated effects of Chinese visceral adiposity index and risk of comorbidity of hypertension and diabetes mellitus.

Paths in the mediation model	β (95%CI)^*^	OR (95%CI)^*^	*p-*value
HTN-DM comorbidity			
The total effect—path c	0.38 (0.27–0.48)	1.45 (1.30–1.62)	<0.0001
The direct effect—path c’	0.12 (0.00–0.24)	1.13 (1.00–1.27)	0.0566
Path a	0.24 (0.22–0.26)		<0.0001
Path b	1.16 (0.97–1.34)	3.18 (2.63–3.83)	<0.0001
The indirect effect—path ab^#^	0.28 (0.22–0.34)	1.32 (1.25–1.40)	<0.0001
HTN or DM			
The total effect—path c	0.33 (0.24–0.42)	1.39 (1.27–1.52)	<0.0001
The direct effect—path c’	0.25 (0.16–0.34)	1.29 (1.17–1.41)	<0.0001
Path a	0.24 (0.22–0.26)		<0.0001
Path b	0.32 (0.18–0.46)	1.38 (1.19–1.59)	<0.0001
The indirect effect—path ab^#^	0.08 (0.04–0.11)	1.08 (1.04–1.12)	<0.0001
HTN			
The total effect—path c	0.31 (0.22–0.39)	1.36 (1.25–1.48)	<0.0001
The direct effect—path c’	0.31 (0.22–0.40)	1.36 (1.24–1.48)	<0.0001
Path a	0.22 (0.20–0.24)		<0.0001
Path b	0.00 (−0.14–0.14)	1.00 (0.87–1.15)	0.9879
The indirect effect—path ab^#^	0.00 (−0.03–0.03)	1.00 (0.97–1.03)	0.9902
DM			
The total effect—path c	0.25 (0.16–0.35)	1.29 (1.17–1.42)	<0.0001
The direct effect—path c’	−0.04 (−0.15–0.07)	0.96 (0.86–1.07)	0.4587
Path a	0.24 (0.22–0.26)		<0.0001
Path b	1.29 (1.11–1.46)	3.62 (3.04–4.31)	<0.0001
The indirect effect—path ab^#^	0.31 (0.26–0.37)	1.36 (1.29–1.44)	<0.0001

TyG, triglyceride-glucose index; OR, odds ratio; CI, confidence interval; HTN, hypertension; DM, diabetes mellitus. Adjusted for age, gender, educational level, marital status, dietary, smoking, drinking, physical activity, family history of HTN or DM, total cholesterol, cardiovascular disease, and cancer.

For HTN or DM, the OR (95%CI) for total (path c), direct (path c’), and indirect (path ab^#^) effects were 1.39 (1.27–1.52), 1.29 (1.17–1.41), and 1.08 (1.04–1.12), which indicated that TyG index partly mediated the association between CVAI and HTN or DM. For DM, significant total (path c), indirect (path ab^#^) effects, and insignificant direct (path c’) effect suggested that TyG index completely mediated the association. However, we failed to observe the mediating effect for TyG index on the association of CVAI with HTN.

## Discussion

The current study first found a linear association between CVAI and HTN-DM comorbidity, and per SD increase was associated with 45% increased risk. Linear associations of CVAI with HTN or DM, HTN, and DM were also reported, and the risk increased 39%, 36%, and 28% for per SD increment. Compared with quartile 1 of CVAI, the risk of HTN-DM comorbidity, HTN or DM, HTN, and DM increased 190%, 125%, 112%, and 96% for quartile 4. In addition, we also found TyG index largely mediating the association between CVAI and HTN-DM comorbidity, partly mediating the association for HTN or DM, and completely mediating the association for DM. Our results provide additional epidemiological evidence for preventing comorbidity or multimorbidity of metabolic disease.

With the improvement of medical care, increasing prevalence of chronic disease, and intensification of aging, comorbidity or multimorbidity has become common (7, 24). In addition, comorbidity or multimorbidity would become more common when treating chronic disease without tackling excess adiposity (25, 26). Consistent with our study, positive associations of CVAI with HTN and DM were reported based on Chinese and Japanese population (10, 12, 27). Different from the above study, we focused on elderly people and explored the association between CVAI and HTN-DM comorbidity and HTN or DM. We found that CVAI was linearly associated with HTN-DM comorbidity, and age difference (<75 vs. ≥75 years) was detected in the association (ORs, 1.32 vs. 2.03). The different effects across age groups may be due to the difference in fat distribution and accumulation, aging process, and other underlying mechanisms (16, 28, 29).

Our mediation analyses showed TyG index playing a key role in the association of CVAI with HTN-DM comorbidity. Similar to our results, a study by Dong et al. also reported TyG index mediating the association between BMI/WC and HTN-DM comorbidity (30). In addition, consistent with our findings for HTN or DM, recent studies found that the BMI-HTN or BMI-DM association was medicated by TyG index (31, 32). Our results also suggested a positive association between CVAI and insulin resistance (TyG index), which was consistent with other studies (33, 34). A recent review described that features induced by obesity including hyperinsulinemia, activation of the sympathetic nervous system, chronic inflammation, and changes in adipokines were potential mechanisms for HTN-DM comorbidity (35). Despite of those pathophysiological bases, some genetic predisposition for obesity may also increase risk of HTN-DM comorbidity (36, 37).

These findings have certain public health implications. Due to the accumulation and heterogeneity of HTN and DM, comorbidity presents a difficult prevention target if approaching these diseases separately (38, 39). Obesity is an important, modifiable, and economical target for disease prevention (39, 40). Considering a different health effect for different quantity and distribution of body fat in different age stages, CVAI, a reliable indicator of VAT, may be a suitable index of obesity to evaluate its health effect (41–43). Because Asian populations have more VAT accumulation at lower BMI values as compared with Western populations, VAI developed in Western population may not reflect AVT well in Chinese adults and has been proven in previous studies (10, 12, 44, 45). Additionally, CVAI is established based on age, BMI, WC, TG, and HDL-C, which are feasible for measuring in routine clinical practice. The measurement of CVAI will benefit identifying high risk of HTN-DM comorbidity, and maintaining healthy lifestyles to reduce CVAI will help to prevent incident HTN-DM comorbidity (46, 47).

Despite its valuable findings and potential public health implications, some limitations should be noted. First, the cross-sectional design cannot provide causal association between CVAI and HTN-DM comorbidity. Second, although we adjusted for various covariates, other confounders, such as environmental pollution and psychological factors, may also affect the association. Third, the study participants were Chinese elderly people, so our findings require validation in other Asian populations. Guidelines focusing on visceral adiposity for different populations should also be developed based on native evidence. Finally, although we conducted the research with strict accordance to the study protocol, potential measurement bias may exist due to the differences of medical staff, measuring environment, and so on.

## Conclusions

Our results showed linear associations of CVAI with HTN-DM comorbidity, HTN or DM, HTN, and DM in Chinese elderly population. TyG index played an important role in the CVAI comorbidity of HTN-DN association. Our findings needed to be validated in prospective cohort studies and clinical trials.

## Data availability statement

The raw data supporting the conclusions of this article will be made available by the authors, without undue reservation.

## Ethics statement

The studies involving human participants were reviewed and approved by Ethics Committee of Huanghuai University. The patients/participants provided their written informed consent to participate in this study.

## Author contributions

YR substantially contributed to the design and drafting of the study and the analysis and interpretation of the data. LC, RQ, and MH revised it critically for important intellectual content. All authors were involved in collecting data and approved the final version of the manuscript.
