# Supplementary material for: Dose–response association of Chinese visceral adiposity index with comorbidity of hypertension and diabetes mellitus among elderly people
Source: Front Endocrinol (Lausanne). 2023 May 12;14:1187381. doi: 10.3389/fendo.2023.1187381 (PMC10213325; doi:10.3389/fendo.2023.1187381)
Supplement: Supplementary file 1 [file Table_1.docx]

**Table S1.** Sensitivity analysis for per SD increase by excluding participants with cardiovascular disease or cancer.

|  | **HTN-DM comorbidity** | **HTN or DM** | **HTN** | **DM** |
| --- | --- | --- | --- | --- |
| **Total** | 1.45 (1.30-1.63) | 1.37 (1.25-1.49) | 1.34 (1.23-1.46) | 1.28 (1.16-1.41) |
| **Gender** |  |  |  |  |
| Men | 1.39 (1.20-1.61) | 1.33 (1.19-1.49) | 1.32 (1.18-1.47) | 1.23 (1.08-1.40) |
| women | 1.49 (1.24-1.78) | 1.46 (1.27-1.68) | 1.42 (1.24-1.62) | 1.30 (1.11-1.53) |
| **Age** |  |  |  |  |
| <75 y | 1.33 (1.17-1.51) | 1.40 (1.26-1.55) | 1.38 (1.25-1.52) | 1.18 (1.06-1.32) |
| ≥75 y | 1.97 (1.54-2.53) | 1.39 (1.15-1.67) | 1.31 (1.09-1.57) | 1.67 (1.34-2.09) |

Data are odds ratios (ORs) and confidence intervals (CIs).

HTN, hypertension; DM, diabetes mellitus.

Adjusted for age, gender, educational level, marital status, dietary, smoking, drinking, physical activity, family history of HTN or DM, and total cholesterol.

**Table S2.** Sensitivity analysis for quartiles of Chinese visceral adiposity index.

|  | **Quartile 1** | **Quartile 2** | **Quartile 3** | **Quartile 4** | ***P* value** |
| --- | --- | --- | --- | --- | --- |
|  | | | | | |
| **HTN-DM comorbidity** | 1.00 | 1.63 (1.15-2.31) | 2.15 (1.51-3.05) | 3.02 (2.15-4.22) | <0.001 |
| **HTN or DM** | 1.00 | 1.27 (1.02-1.59) | 1.60 (1.26-2.02) | 2.11 (1.65-2.69) | <0.001 |
| **HTN** | 1.00 | 1.37 (1.10-1.69) | 1.66 (1.32-2.08) | 2.03 (1.61-2.56) | <0.001 |
| **DM** | 1.00 | 1.17 (0.88-1.55) | 1.44 (1.08-1.92) | 1.96 (1.49-2.59) | <0.001 |

Data are odds ratios (ORs) and confidence intervals (CIs).

HTN, hypertension; DM, diabetes mellitus.

Adjusted for age, gender, educational level, marital status, dietary, smoking, drinking, physical activity, family history of HTN or DM, and total cholesterol.
